# EFFECTS OF ENVIRONMENTAL EXPOSURE ON DISPARITIES IN THE RISK OF ALZHEIMER’S DISEASE AND RELATED DEMENTIAS

**DOI:** 10.1093/geroni/igae098.1134

**Published:** 2024-12-31

**Authors:** Igor Akushevich, Arseniy Yashkin, Vito Di Bona, Julia Kravchenko

**Affiliations:** Duke University, Durham, North Carolina, United States; Duke University, Durham, North Carolina, United States; Duke University, Durham, North Carolina, United States; Duke University School of Medicine, Durham, North Carolina, United States

## Abstract

Chronic exposures to a range of environmental contaminants, in particular fine particulate matter (PM2.5), have been shown to be associated with an increased risk of Alzheimer’s disease and related dementia (AD/ADRD). However, causal relationships between the effects of ambient air pollutants (especially for low-to-moderate-level exposures) and PM2.5 constituents on AD/ADRD risk remain understudied in both the general population and vulnerable population groups in the U.S. The objective of this study is to evaluate the effects of exposures to PM2.5 and PM2.5 constituents (including black carbon, NH4, SO4, trace elements, and some others), test the causality of contaminant-outcome associations, and explain the epidemiological mechanisms of these causal pathways. To address this objective, we used Medicare-claims data and zip-code-level environmental data provided by the Socioeconomic Data and Applications Center (SEDAC) including daily and annual estimates of the concentrations of PM2.5 for 2000-2016, and trace elements including bromine, calcium, copper, aluminum, iron, lead, nickel, potassium, silicon, vanadium, and zinc. We reconstructed the concentration-response patterns of exposures to ambient contaminants on AD/ADRD risk and applied the parametric g-formula approach to test the causality of detected effects. We evaluated the concentration-response functions for all contaminants and found deviations from the linear dependence for concentrations above 10 µg/m3 (WHO standards) and for the Hispanic subpopulation. The highest risks were found for Native Americans, with a hazard ratio (HR) vs. White subpopulation exceeding HR>3 over the full range of average concentrations (5-12 µg/m3 for PM2.5). NH4, SO4 and lead demonstrated the highest effect among constituents of PM2.5.

